# Efficient Drug Delivery of Paclitaxel Glycoside: A Novel Solubility Gradient Encapsulation into Liposomes Coupled with Immunoliposomes Preparation

**DOI:** 10.1371/journal.pone.0107976

**Published:** 2014-09-29

**Authors:** Tsukasa Shigehiro, Tomonari Kasai, Masaharu Murakami, Sreeja C. Sekhar, Yuki Tominaga, Masashi Okada, Takayuki Kudoh, Akifumi Mizutani, Hiroshi Murakami, David S. Salomon, Katsuhiko Mikuni, Tadakatsu Mandai, Hiroki Hamada, Masaharu Seno

**Affiliations:** 1 Department of Biotechnology, Graduate School of Natural Science and Technology, Okayama University, Okayama, Japan; 2 Mouse Cancer Genetics Program, Center for Cancer Research, National Cancer Institute, Frederick, Maryland, United States of America; 3 Ensuiko Sugar Refining Co., Ltd., Tokyo, Japan; 4 Faculty of Life Science, Kurashiki University of Science and the Arts, Kurashiki, Japan; 5 Faculty of Science, Okayama University of Science, Okayama, Japan; Cornell University, United States of America

## Abstract

Although the encapsulation of paclitaxel into liposomes has been extensively studied, its significant hydrophobic and uncharged character has generated substantial difficulties concerning its efficient encapsulation into the inner water core of liposomes. We found that a more hydrophilic paclitaxel molecule, 7-glucosyloxyacetylpaclitaxel, retained tubulin polymerization stabilization activity. The hydrophilic nature of 7-glucosyloxyacetylpaclitaxel allowed its efficient encapsulation into the inner water core of liposomes, which was successfully accomplished using a remote loading method with a solubility gradient between 40% ethylene glycol and Cremophor EL/ethanol in PBS. Trastuzumab was then conjugated onto the surface of liposomes as immunoliposomes to selectively target human epidermal growth factor receptor-2 (HER2)-overexpressing cancer cells. *In vitro* cytotoxicity assays revealed that the immunoliposomes enhanced the toxicity of 7-glucosyloxyacetylpaclitaxel in HER2-overexpressing cancer cells and showed more rapid suppression of cell growth. The immunoliposomes strongly inhibited the tumor growth of HT-29 cells xenografted in nude mice. Notably, mice survived when treated with the immunoliposomes formulation, even when administered at a lethal dose of 7-glucosyloxyacetylpaclitaxel *in vivo*. This data successfully demonstrates immunoliposomes as a promising candidate for the efficient delivery of paclitaxel glycoside.

## Introduction

Taxanes, such as paclitaxel (PTX) and docetaxel, comprise one of the most effective classes of anticancer drugs that function by stabilizing microtubules and inhibiting the cell cycle [1], [2]. PTX has been used for the treatment of breast, ovarian, colon, brain, and non-small cell lung cancers. Because of the significant hydrophobicity of PTX, the commercial formulation, Taxol, contains the surfactant Cremophor EL and ethanol [1]. Since the solvent is considered as the cause of the reported side effects of Taxol [3], an alternative PTX formulation without the solvent has been sought. Nanoparticles such as liposomes and micelles are thought useful for producing PTX formulations that do not cause the side effects associated with Cremophor EL and ethanol [1], [4].

Liposomal drug delivery has been used to improve the therapeutic effect of drugs in tumor cells by preventing significant side effects in normal cells. Liposomes of particle size ranging from 100 to 200 nm can accumulate in solid tumors by enhanced permeability and retention (EPR) effects because of the presence of abnormal and leaky blood vessels and impaired lymphatic drainage associated with solid tumors [5]. Compared with non-targeting liposomes, active-targeting liposomes that involve the use of attached specific ligands more effectively enhance the anticancer activity of the encapsulated drug, as ligand-mediated endocytosis helps facilitate the uptake of the actively delivered drug [6]. Human epidermal growth factor receptor-2 (HER2) is a 185 kDa type I receptor tyrosine kinase glycoprotein that is often overexpressed in breast, ovarian, colon, and lung cancer tissues [7]. HER2 overexpression in human breast cancer cells increases their metastatic potential and is associated with a poor prognosis. Therefore, HER2 has been considered as a target for cancer therapy [8]. Liposomal drug delivery targeting HER2 has been well studied, and immunoliposomes displaying anti-HER2 antibodies such as trastuzumab exhibit greater anticancer activity than non-targeting liposomes by specifically targeting HER2-overexpressing cancer cells [9]–[12].

Several attempts are currently underway to encapsulate PTX into liposomes. PTX is too hydrophobic to be encapsulated into the inner water core of liposomes and in previous studies PTX has been inserted into the hydrophobic space of a lipid bilayer to generate active liposomes constructs [13]–[17]. However, to prepare stable liposomes with significant amounts of drug, PTX should be encapsulated into the inner water core of liposomes instead of inserted into the lipid bilayer. In the case of Doxil, doxorubicin is encapsulated into the inner water core of liposomes using a unique procedure of remote loading with an ammonium sulfate gradient [18]. A similar remote loading approach could be applied to achieve efficient encapsulation of PTX into the inner water core of liposomes.

In this paper, we demonstrate that glycosylated PTX (7-glucosyloxyacetylpaclitaxel; gPTX), which is a more hydrophilic PTX derivative with a glucose moiety coupled at 7-OH of PTX [19], is more efficiently encapsulated into the inner water core of liposomes than PTX. We also report encapsulation of sufficient amounts of gPTX using a novel remote loading method with a solubility gradient allowing high efficiency. Furthermore, targeted drug delivery was evaluated by constructing immunoliposomes with trastuzumab.

## Materials and Methods

### Materials

Dipalmitoylphosphatidylcholine (DPPC), 1,2-distearoyl-*sn*-glycerol-3-phosphoethanolamine-*N*-[methoxy (polyethylene glycol)-2000] (mPEG–DSPE), and 1,2-distearoyl-*sn*-glycerol-3-phosphoethanolamine-*N*-[maleimide (polyethylene glycol)-2000] (Mal–PEG–DSPE) were obtained from NOF Co. (Tokyo, Japan). Cholesterol (Chol) was purchased from Kanto Chemical Co., Inc. (Tokyo, Japan). Thiazolyl blue tetrazolium bromide (MTT), RPMI 1640 medium and DMEM were obtained from Sigma-Aldrich (St Louis, MO, USA). Trastuzumab was a generous gift from Chugai Pharmaceutical. CO., LTD. (Tokyo, Japan). gPTX was synthesized as previously described [19].

### Cell culture

The human colon cancer cell line HT-29 (ATCC, Manassas, VA, USA) and the human breast cancer cell line SK-BR-3 (ATCC) were cultured in RPMI 1640 medium supplemented with 10% fetal bovine serum (FBS) and 100 µg/mL kanamycin. The human breast cancer cell line MDA-MB-231 (ATCC) was cultured in DMEM supplemented with 10% FBS and 100 µg/mL kanamycin. Cells were maintained at 37°C in an atmosphere of 5% CO_2_.

### Evaluation of gPTX solubility

The difference in hydrophilicity between gPTX and PTX was evaluated using a C_18_ reverse-phase HPLC column (GL Sciences Inc., Tokyo, Japan) under isocratic elution with 55% (v/v) methanol at a flow rate of 1 mL/min. PTX or gPTX at 5 µg/mL was injected at a volume of 10 µL and detected at 227 nm.

The maximum solubility of PTX and gPTX in 40% (v/v) ethylene glycol (EG) was determined as follows. First, 2 mg of PTX or gPTX were dissolved in 1 mL of 40% EG followed by three rounds of sonication for 10 min. The solution was then centrifuged at 12,000×*g* for 20 min and the supernatant was analyzed for the concentration by reverse-phase HPLC under isocratic elution with 60% (v/v) methanol at a flow rate of 1 mL/min.

The maximum solubility of PTX or gPTX in Cremophor EL/ethanol/PBS (12∶12∶76 volume ratio) (CEP) was determined by gelation after adding different amounts of PTX or gPTX in 1 mL of CEP.

### Tubulin polymerization assay

The stability of tubulin polymerization was evaluated using the Tubulin Polymerization Assay Kit (Cytoskeleton Inc., Denver, USA) according to the manufacturer's instructions. In brief, 2 mg/mL porcine tubulin in tubulin polymerization buffer containing 80 mM PIPES pH 6.9, 2.0 mM MgCl_2_, 0.5 mM EDTA, 1.0 mM GTP, and 10 µM fluorescent reporter was prepared. Then tubulin solution was transferred to a prewarmed 96-well plate that contained 3 µM PTX or gPTX. The polymerization of tubulin was monitored as fluorescence at 37°C for 60 min, and the reading speed was programmed at 1 cycle/min with excitation and emission wavelengths of 360 and 420 nm, respectively, using the MTP-800 microplate reader (Corona Electric, Ibaraki, Japan). The stability of polymerization was judged by the differences of fluorescence intensities in the presence or absence of PTX or gPTX. The experiment was independently performed in triplicate and the mean and standard deviation (S.D.) of the fluorescent intensities were calculated.

### Preparation of liposomes containing PTX/gPTX

Liposomes composed of DPPC and Chol with 5 mol% mPEG–DSPE were prepared by the thin-film hydration method. In brief, DPPC and Chol with 5 mol% mPEG–DSPE were dissolved in an organic solvent of chloroform/methanol (9∶1 v/v) in an egg flask. The flask was connected to a rotary evaporator, which was maintained at 45°C under aspirator vacuum. The resulting lipid film was left overnight under vacuum to remove remaining organic solvent. The completely dehydrated lipid film was suspended in CEP by vortexing at 60°C, resulting in the formation of multilamellar vesicles (MLVs). MLVs were sonicated twice by the Sonicator 3000 (Misonix, NY, USA) equipped with 3.2 mm micro tip for 5 min at 60°C to form small lamellar vesicles (SLVs). The outer solvent of the liposomes was replaced CEP with PBS by ultrafiltration with a 100K-membrane filter (Merck Millipore Ltd., Billerica, USA) at 12,000×*g* for 20 min for five times. Then, PTX (0.1 mg/mL) or gPTX (1 mg/mL) in 40% EG was added into the solution of liposome encapsulating CEP (CEP-L) at 60°C. Thus, PTX- or gPTX-containing liposomes (PTX- or gPTX-L) were prepared under the solubility gradient, which is a remote loading method. The initial ratio of the PTX or gPTX to initial lipids/Chol at the remote loading was 0.005 or 0.05 (w/w). PTX- or gPTX-L was then concentrated to the volume before added drug by ultrafiltration. This encapsulation process was conducted three times. Finally, residual PTX or gPTX was removed by washing the liposomes with PBS followed by ultrafiltration at 12,000×*g* for 20 min for five times.

For the direct encapsulation, the lipid film was suspended in CEP-dissolved PTX or gPTX at a concentration of 0.3 or 3 mg/mL with the initial ratio of PTX or gPTX to initial lipids/Chol 0.016 or 0.16 (w/w) as described above for the preparation of CEP-L. The drug-encapsulating MLVs were further sonicated to prepare SLVs, and the residual drug was removed by washing the liposomes with PBS as described above.

### Evaluation of the influence of lipid compositions and incubation time for encapsulation

The influence of different molar ratios of DPPC to Chol of 3∶1, 3∶2, and 3∶3 was evaluated in the solubility gradient method. To encapsulate the drug, 1 mg/mL gPTX in 40% EG and CEP-L were incubated for 30 min at 60°C. The encapsulation efficiency (EE) was assessed by HPLC. The stability of gPTX-L in different lipid compositions was evaluated in RPMI 1640 medium supplemented with 10% FBS at 37°C. Drug retention according to the time course was assessed by HPLC. The influence of the incubation time was evaluated when the molar ratio of DPPC to Chol was 3∶1.

### Preparation of immunoliposomes containing gPTX

To prepare HER2-targeting immunoliposomes containing gPTX (gPTX-IL), gPTX-L displaying trastuzumab on the surface of the liposome was prepared. CEP-L composed of DPPC and Chol with 4 mol% mPEG–DSPE were incubated with 1 mol% Mal–PEG–DSPE at 50°C for 10 min to introduce maleimide functional groups to conjugate antibodies [20]. Then, gPTX was encapsulated using the solubility gradient method described above. To immobilize antibody on the surface of the liposomes, SH groups were introduced into trastuzumab by treatment with 2-iminothiolane at a molar ratio of 1∶50 in 25 mM HEPES, pH 8.0 containing 140 mM NaCl. The mixture was subsequently incubated for 1 h at room temperature in the dark [20], [21]. After purification by gel chromatography with a G25 PD-10 column (GE Healthcare, Tokyo, Japan), trastuzumab was incubated with liposomes containing Mal–PEG–DSPE overnight at 4°C. Free trastuzumab was removed by ultrafiltration with a 300K-membrane filter (Sartorius Stedim Biotech GmbH, Gottingen, Germany) at 6000×*g* for 20 min.

### Evaluation of Encapsulation efficiency (EE) and loading efficiency (LE)

EE was calculated as the ratio of the amount of PTX/gPTX encapsulated into liposomes to the initial amount of the drug. LE was calculated as the molar ratio of the drug encapsulated into liposomes to the total of lipids and Chol. The amount of encapsulated drug was evaluated by C_18_ reverse-phase HPLC under an isocratic condition of 60% (v/v) methanol at a flow rate of 1 mL/min. Ten µL of each sample were injected and the drug was detected at 227 nm.

### Particle size and zeta potential

The particle size and zeta potential of liposomes were determined by dynamic light scattering and electrophoretic light scattering with an ELS-8000 microscope (Otsuka Electronics, Osaka, Japan).

### Transmission electron microscope (TEM) image of PTX-L and gPTX-L

The liposome samples were negatively stained with tungsten phosphate, and observed with a TEM (H-7600, Hitachi, Tokyo, Japan) at an accelerating voltage of 100 kV. The TEM study was conducted by Hanaichi Ultra-Structure Research Institute (Aichi, Japan).

### Evaluation of concentration-dependent cytotoxic effects

*In vitro* cytotoxicity was evaluated by the MTT assay after 72 h of drug exposure. HT-29, SK-BR-3 and MDA-MB-231 cells were seeded in a 96-well plate at 5.0×10^3^ cells/well. After incubation at 37°C in 5% CO_2_ for 24 h, different concentrations of PTX or gPTX were added to each well. After incubation for 72 h, 5 mg/mL MTT solution was added at a final concentration of 0.6 mg/mL in each well and the plate was incubated for 4 h. Formed formazan crystals were dissolved in 10% (w/v) SDS with 0.02 N HCl and incubated overnight. Finally, the absorbance of each well was measured at 570 nm using an MTP-800 microplate reader. The experiment was independently performed in triplicate. The concentrations at which cell growth was inhibited by 50% (IC_50_) and 100% (IC_100_) were estimated from the survival curve.

### Evaluation of time-dependent cytotoxic effects

Time-dependent cellular cytotoxicity was evaluated by the MTT assay with drugs at IC_100_. HT-29, SK-BR-3 and MDA-MB-231 cells were seeded in a 96-well plate at 5.0×10^3^ cells/well. After incubation at 37°C in 5% CO_2_ for 24 h, drugs at their IC_100_ were added to each well and incubated for 1, 2, 6, 12, 24, 48, and 72 h. After each round of drug exposure, the medium was replaced with fresh medium without drugs and the incubation was continued for an additional 72 h after the drugs were added. Cell viability was determined by MTT assay. The time required for 50% growth inhibition (IT_50_) was estimated from the survival curve.

### Animal experiments

Animal experimental protocols were reviewed and approved by the ethics committee for animal experiments of Okayama University under the IDs OKU-2012203 and OKU-2014156.

### Evaluation of acute toxicities of drugs *in vivo*

The acute toxicities of the drugs were evaluated with female BALB/c mice (Charles River, Kanagawa, Japan). Mice were bred at 23°C and fed with sterilized food and water. Naked gPTX. gPTX-L and gPTX-IL at the dose of 150 mg/kg gPTX were intravenously injected into 6-week-old mice via tail vein twice in 3 h. As a control, the equivalent amount of CEP contained naked gPTX and the equivalent amount of PBS contained in gPTX-IL was injected into mice. The body weights were monitored for 2 weeks. Mice with more than 20% body weight loss were sacrificed for humane reasons.

### Evaluation of the antitumor effects of drugs *in vivo*

To prepare tumor-bearing mice, 3×10^6^ HT-29 cells were subcutaneously injected into 5-week-old female BALB/c-nu/nu mice (Charles River, Kanagawa, Japan). Mice were bred at 23°C and fed with sterilized food and water. When tumor volumes reached 50–200 mm^3^, mice were randomly divided into several groups, with three or four mice in each group. Naked gPTX, gPTX-L, gPTX-IL, mixture of gPTX-L and trastuzumab, trastuzumab only, CEP only, immunoliposomes encapsulating CEP only (CEP-IL), or PBS was intravenously injected via the tail vein twice in 3 h. The total injected dose of naked gPTX was 100 mg/kg. gPTX-L was injected at the gPTX-equivalent dose of 150 mg/kg. gPTX-IL was injected at the gPTX-equivalent dose of 10 to 150 mg/kg. As the control, the equivalent amount of CEP contained in the naked gPTX at the dose of 100 mg/kg was injected into mice. CEP-IL was also injected as the control at a concentration at which the amount of lipid equivalent to that contained in gPTX-IL at the gPTX-equivalent dose of 150 mg/kg. Trastuzumab (150 mg/kg) was injected at the same concentration of trastuzumab conjugated to gPTX-IL at the gPTX-equivalent dose of 150 mg/kg. Tumor volumes and body weights were measured at 3-day intervals. The tumor volume was calculated using the formula 0.5× width^2^ × length, in which width is the smallest diameter and length is the longest diameter. The mice were sacrificed at day 30 and the tumors were weighed.

The antitumor effect by repeated administration was evaluated in HT-29 cell-bearing BALB/c-nu/nu mice. Mice were randomly divided into several groups, with for mice in each group. The mice treated with gPTX-L, gPTX-IL, mixture of gPTX-L and trastuzumab, CEP-L, CEP-IL, trastuzumab, or PBS were intravenously injected via the tail vein 3 times at day 0, 10 and 20 with the gPTX-equivalent dose of 150 mg/kg at each time. Tumor volumes and body weights were measured at 3 or 4-day intervals. Mice with more than 20% body weight loss or tumors larger than 2,500 mm^3^ were sacrificed for humane reasons.

### Statistical analysis

The results are presented as the mean ± S.D. where necessary. Statistical analyses were performed using Student's *t*-test. *P*<0.05 was considered statistically significant.

## Results

### Characterization of gPTX

**Figure 1:**
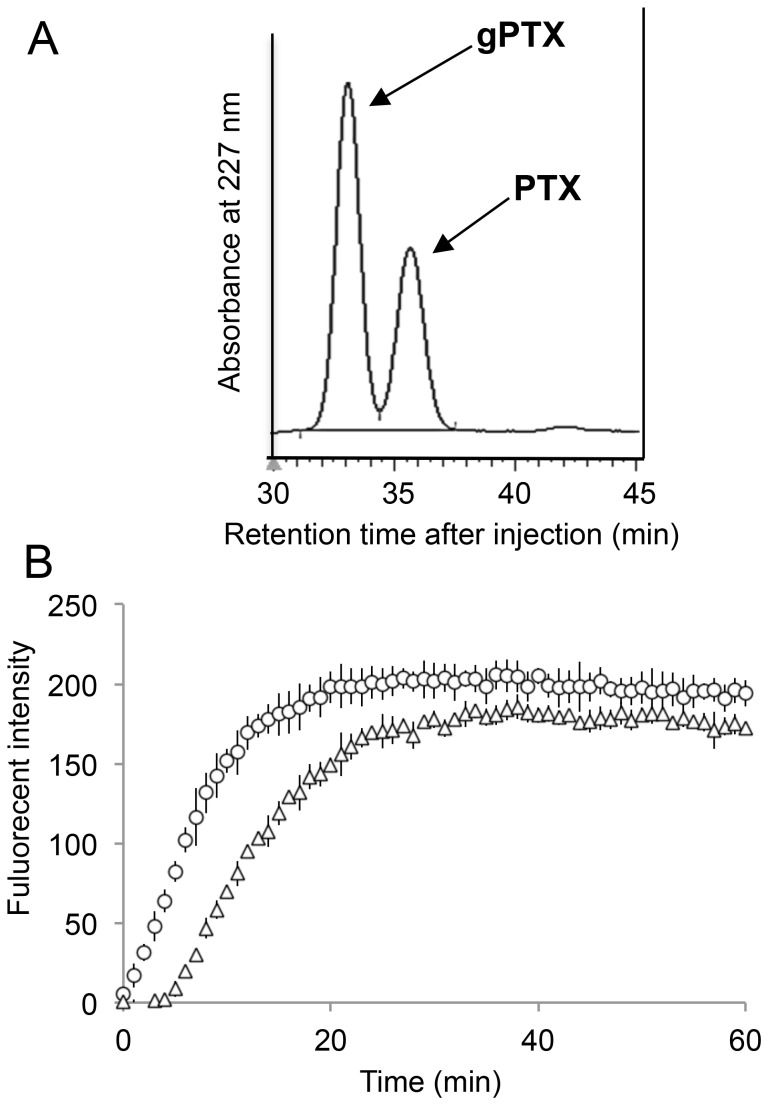
Characterizations of gPTX. A, The hydrophobicity of PTX and gPTX was evaluated by reverse-phase HPLC using a C_18_ column at a flow rate of 1 mL/min with 55% (v/v) methanol under an isocratic condition. B, The ability of 3 µM PTX (open circle) and gPTX (open triangle) to stabilize porcine tubulin polymerization was evaluated using a Tubulin Polymerization Assay Kit (Cytoskeleton). The fluorescent reporter was detected with excitation at 360 nm and emission at 420 nm. Each dot represents the mean ± S.D. (n = 3).

The hydrophilicity of gPTX was evaluated by C_18_ reverse-phase HPLC (Fig. 1A). The retention time of gPTX was 33.3 min whereas that of PTX was 35.9 min, indicating that gPTX was more hydrophilic than PTX. The hydrophilicity was further assessed by evaluating solubility in 40% EG and CEP. The maximal solubility of gPTX was 1.10 mg/mL in 40% EG and 20.0 mg/mL in CEP, whereas that of PTX was 0.12 mg/mL in 40% EG and 2.0 mg/mL in CEP. These results indicate that gPTX had a 10-fold higher solubility than PTX in these solvents. These data are summarized in Table 1.

**Table 1:** Solubility of PTX and gPTX in different solvents.

Solvent	Max. solubility (mg/mL)	
	PTX	gPTX
*H_2_O	0.4×10^−3^	23×10^−3^
40% EG	0.12	1.10
**CEP	2.0	20.0

* *Solubility of H_2_O was referred as described previously* [19].

** *CEP consists of Cremophor EL and ethanol in PBS (12∶12∶76 volume %)*.

Since the mechanism of PTX anticancer activity is based on the inhibition of tubulin depolymerization, gPTX tubulin stabilization activity was compared with that of PTX (Fig. 1B). gPTX induced slower polymerization than PTX and the maximal tubulin polymerization mediated by gPTX was 89.9±4.1% of that induced by PTX. This result suggests that gPTX exhibits almost equivalent inhibition of tubulin depolymerization, although the affinity of gPTX for β-tubulin may be lower than that of PTX because of the glucose moiety.

### Preparation and characterization of gPTX-L and gPTX-IL

To encapsulate large amounts of gPTX into the inner water core of liposomes, we first attempted a direct encapsulation approach. In this method, the thin-film lipid layer was rehydrated with the solvent-dissolved gPTX at the maximal concentration. Since observed EE and LE were less than 1% and 0.1%, respectively, gPTX at the maximal concentration in 40% EG (1 mg/mL) was found inadequate for effective encapsulation (Table S1). In contrast, we found CEP more useful as the solvent for gPTX encapsulation than 40% EG. CEP-dissolved gPTX at the maximal concentration (20 mg/mL) showed 17.6% of EE and 13.7% of LE. However, the EE obtained by direct encapsulation with CEP is still less than 20%, and an EE of more than 60% is required for clinical application.

**Figure 2:**
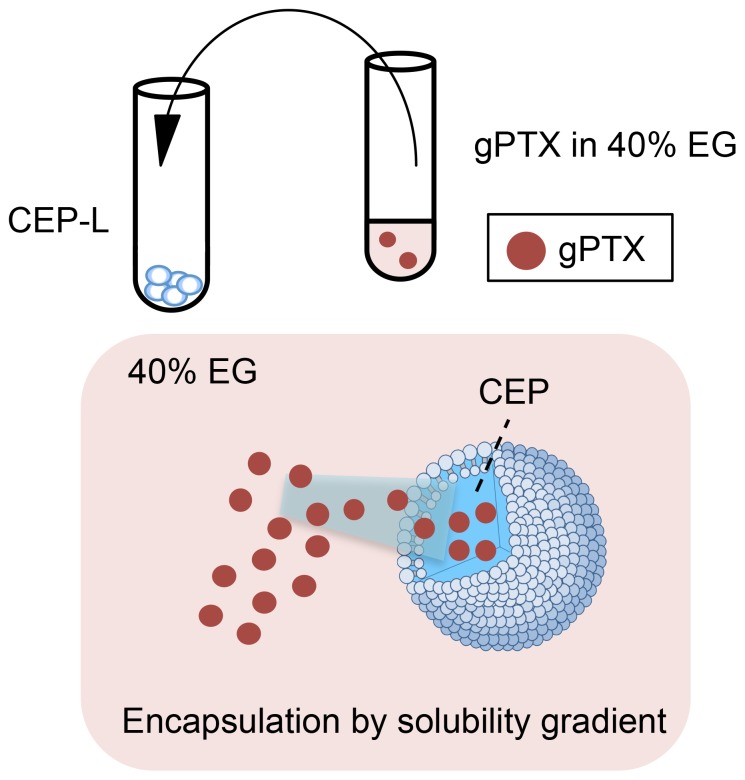
Schematic illustration of remote loading with a solubility gradient. CEP-encapsulated liposomes (CEP-L) and gPTX dissolved in 40% EG were mixed and incubated. gPTX was encapsulated into liposomes under a solubility gradient.

**Figure 3:**
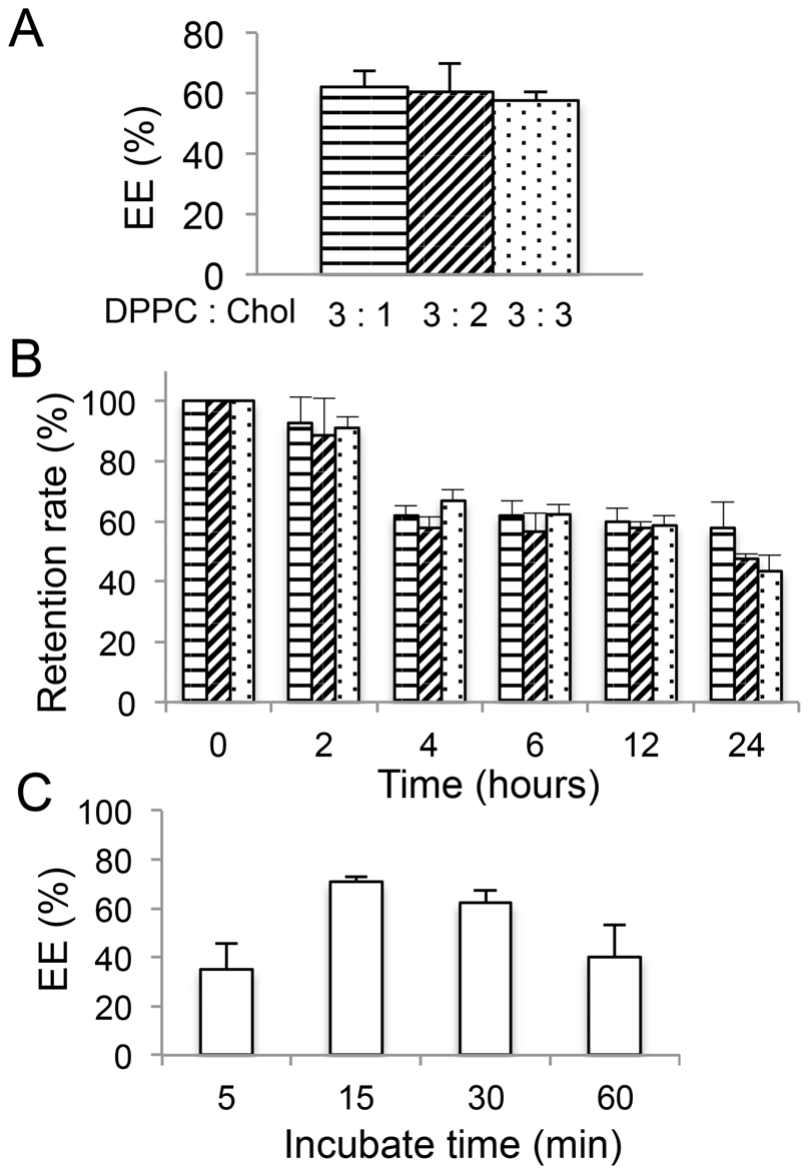
Influence of the lipid composition and incubation time for drug encapsulation by the solubility gradient method. A, The encapsulation efficiency (EE) of gPTX-L with various lipid compositions was evaluated after 30 min of incubation for gPTX encapsulation. B, The drug retention of gPTX-L with different lipid compositions was evaluated in medium supplied with 10% FBS at 37°C. C, The efficiency of drug encapsulation under different durations of incubation was evaluated with a DPPC to Chol ratio of 3∶1.

To achieve more efficient encapsulation, we evaluated a remote loading strategy with a solubility gradient between 40% EG and CEP (Fig. 2). To optimize the lipid composition for gPTX-L, different DPPC/Chol ratios were assessed by remote loading under the solubility gradient. DPPC:Chol at a molar ratio of 3∶1 appeared suitable in regards to EE and retention rate in 10% FBS at 37°C (Fig. 3A, B). We next evaluated various incubation times at 60°C for the encapsulation of gPTX under these parameters, and found that 15 min of incubation achieved the highest EE (Fig. 3C). Thus, liposomes composed of DPPC and Chol at a molar ratio 3∶1 with 5 mol% mPEG–DSPE and an incubation period of 15 min for drug encapsulation were used for the subsequent experiments.

**Figure 4:**
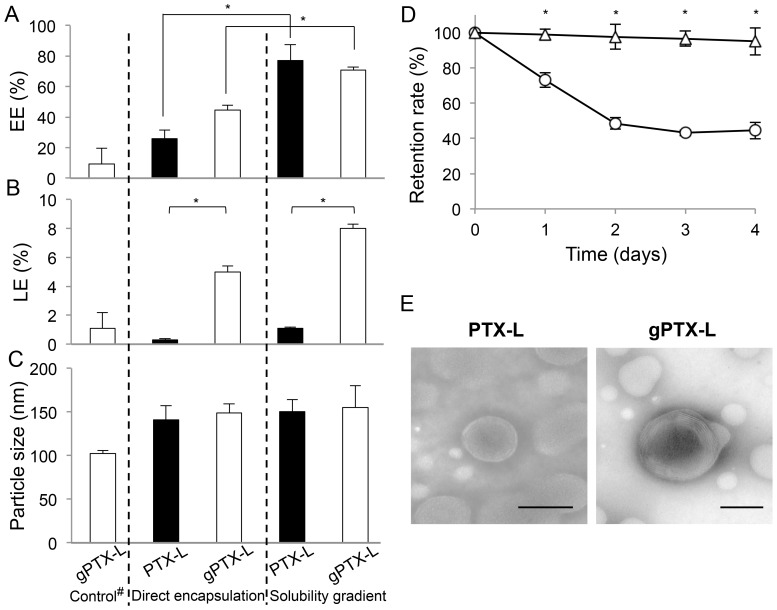
Comparison of the encapsulation efficiency (EE), loading efficiency (LE), and particle size. A, EE. B, LE. C, particle size. ^#^Control indicated that gPTX-L was prepared with liposomes encapsulating 40% EG in 1 mg/mL gPTX. Comparison of stability at 4°C in PBS with PTX-L and gPTX-L prepared by the solubility gradient method. D, The retention rates of PTX-L (open circle) and gPTX-L (open triangle) were evaluated by HPLC. E, TEM images of PTX-L and gPTX-L. Each formulation was observed after 4 days of incubation. Each data point is presented as the mean ± S.D. (n = 3). *, *P*<0.05.

Encapsulation of gPTX under these conditions by remote loading showed 70.8% of EE and 8.0% of LE, whereas direct encapsulation in CEP showed 44.6% of EE and 5.0% of LE (Fig. 4A, B). The observed diameter of gPTX-L was almost equivalent in both encapsulation methods, at approximately 140 to 150 nm (Fig. 4C). The EE of PTX by the solubility gradient method was almost equivalent to that of gPTX. They exhibited liposomal formulations in TEM images (Fig. 4E). However, the LE of gPTX-L was 8-fold higher than that of PTX-L because of the higher solubility of gPTX compared to that of PTX. Furthermore, PTX-L was unstable in PBS at 4°C, exhibiting a retention rate of drug as low as 44.4% in 4 days, whereas gPTX-L was stable during the same period after the preparation of liposomes (Fig. 4D). In addition, gPTX-L was stable enough to exhibit a retention rate of 97.6% without significant change in particle size (±5.3 nm) after 4 weeks of incubation in PBS at 4°C.

To confer gPTX-L with active targeting potential, trastuzumab was conjugated onto the surface of liposomes to prepare gPTX-IL. The mean particle size of gPTX-IL was approximately 150 nm, which is the optimal size for solid tumor targeting based on EPR effects. The zeta potentials of gPTX-L and gPTX-IL were −3.4±2.9 mV and −3.7±1.3 mV, respectively. The liposomal formulation of gPTX-IL was also observed in TEM image (Fig. S1).

### Cytotoxicity of gPTX, gPTX-L and gPTX-IL *in vitro*

**Figure 5:**
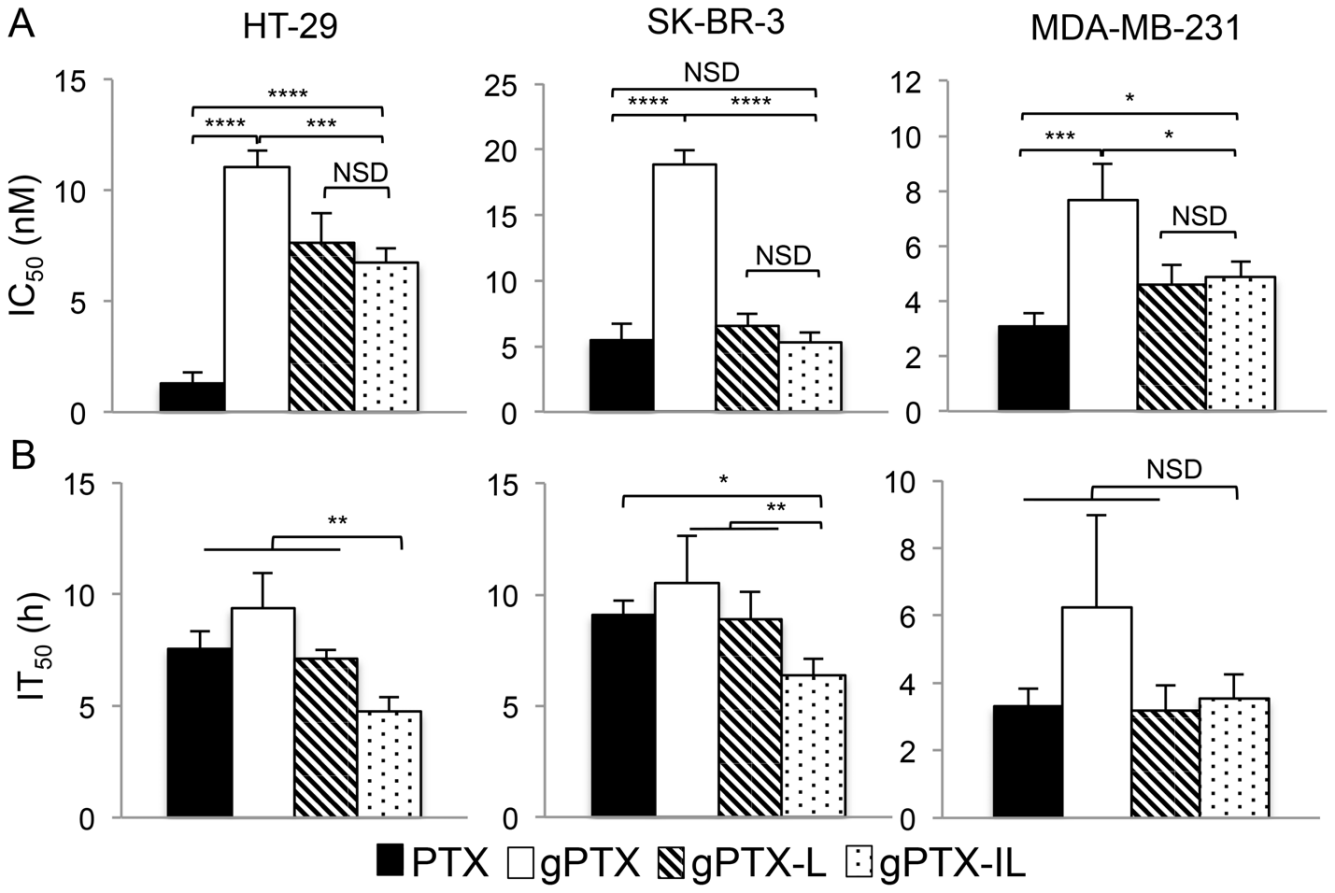
Cytotoxicity of different gPTX formulations by the MTT assay. A, The IC_50_ values after drug exposure for 72 h are shown. B, The IT_50_ values at IC_100_ are shown. The data are presented as the mean ± S.D. for three independent experiments. *, *P*<0.05. **, *P*<0.01. ***, *P*<0.005. ****, *P*<0.001. NSD, no significant difference.

We next evaluated the cytotoxicity of different formulations of gPTX in two HER2-overexpressing cancer cell lines, HT-29 and SK-BR-3, and in the HER2 low-expressing cancer cell line MDA-MB-231 by the MTT assay [7], [22]. IC_50_ and IC_100_ were evaluated after 72 h of exposure to the drugs (Fig. 5A and Table S2). gPTX-L and gPTX-IL exhibited enhanced IC_50_ compared to gPTX, while gPTX-IL exhibited a similar IC_50_ as gPTX-L independent of HER2 expression (Fig. 5A). When IT_50_ was evaluated at IC_100_, the shortest IT_50_ of gPTX-IL was observed following the treatment of HER2-overexpressing HT-29 and SK-BR-3 cells, whereas IT_50_ was not different between gPTX-L and gPTX-IL in the treatment of HER2 low-expressing MDA-MB-231 cells (Fig. 5B and Table S2). These results suggested that gPTX-IL targeted HER2-expressing cells more efficiently than gPTX-L together with the feasible cytotoxicity mediated by the liposomal formulation.

### Acute toxicity of gPTX, gPTX-L and gPTX-IL *in vivo*

**Figure 6:**
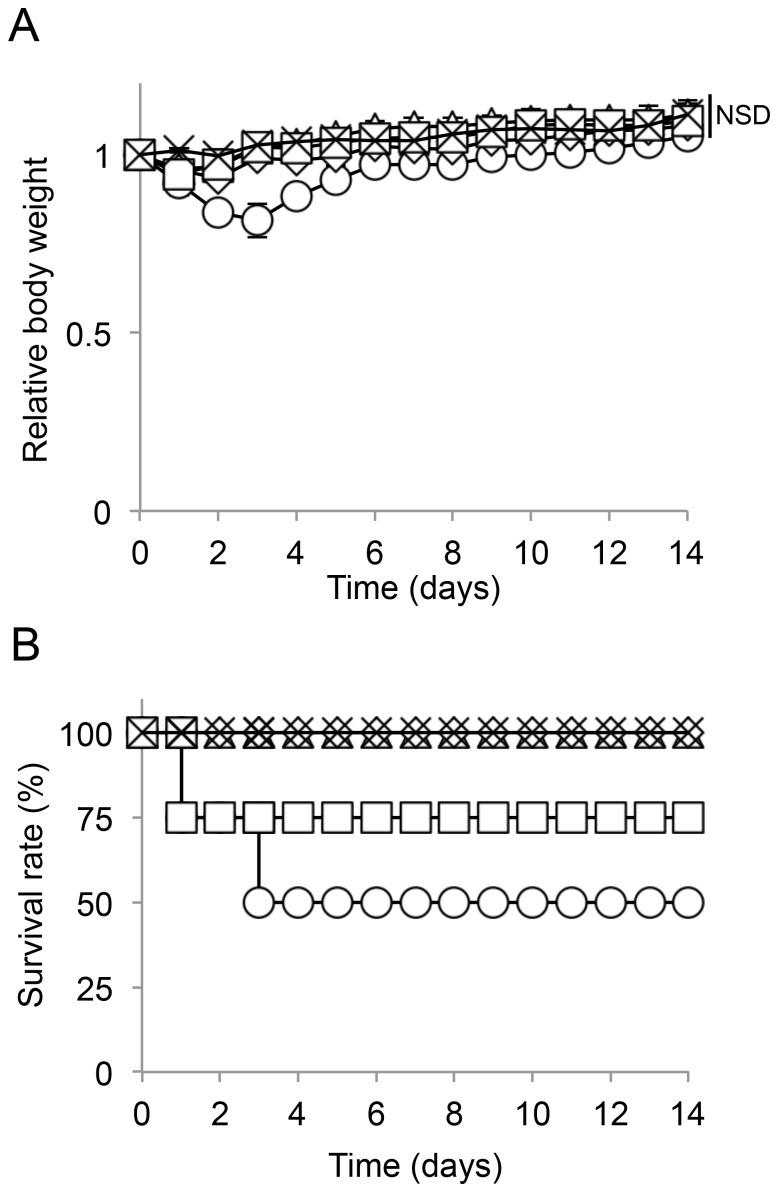
Acute toxicity of the drugs *in vivo*. gPTX (open circle), gPTX-L (open triangle) or gPTX-IL (open diamond) at a concentration of 150 mg/kg, CEP (open square), or PBS (cross) was intravenously injected into 6-week old female BALB/c mice. A, Changes in body weight. B, Survival rate. Data are presented as the mean ± S.D. (n = 4). gPTX is not shown S.D. after day 3. NSD, no significant difference.

Acute toxicity and the lethal dose of gPTX were next evaluated in BALB/c mice (Fig. 6 and S2). The lethal dose of naked gPTX was 150 mg/kg, with a survival rate of 25 to 50%. The survival rate of mice treated with the amount of CEP required to dissolve the lethal dose of gPTX was 50 to 75%. However, 150 mg/kg of gPTX in the liposomal formulation did not exhibit any toxicity, even when gPTX dissolved in CEP was encapsulated into liposomes. Naked gPTX induced a significant loss of body weight for more than a week after injection, whereas gPTX-L and gPTX-IL did not significantly affect body weight.

### Suppression of tumor growth by gPTX-IL *in vivo*

**Figure 7:**
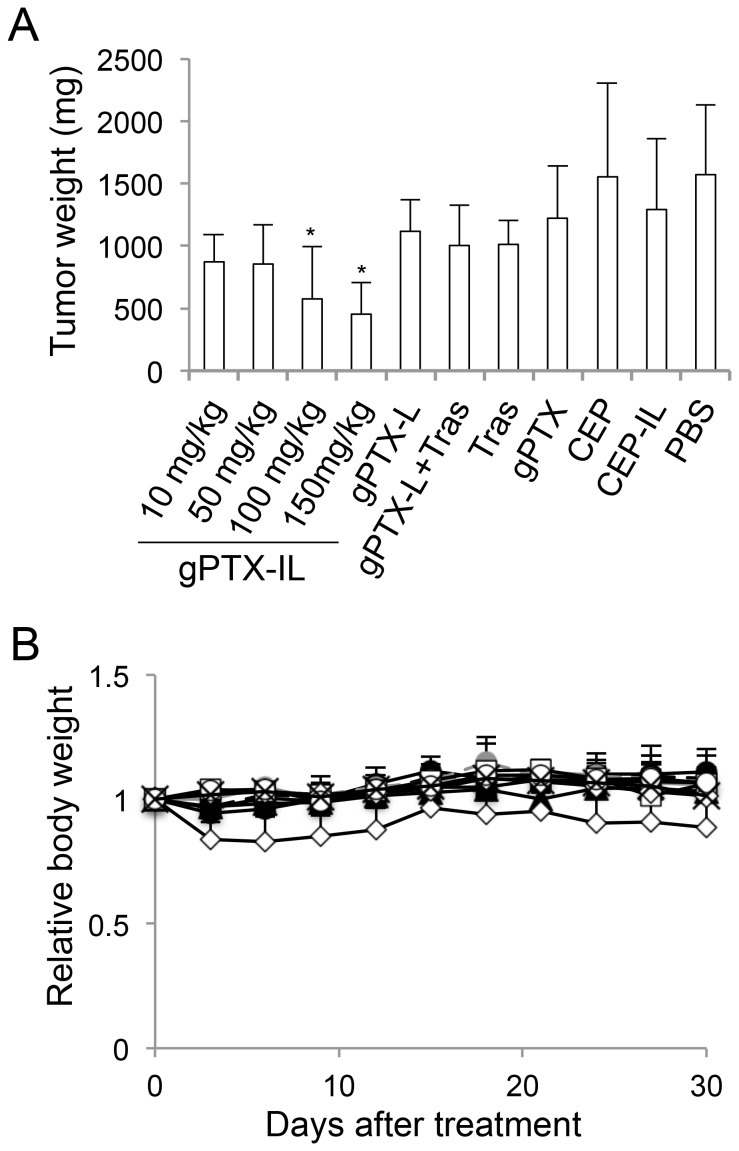
Anticancer efficacy of different gPTX formulations with single administration in HT-29 cells tumor-bearing mice. When the tumor volume reached 50–200 mm^3^, gPTX-IL at the dose of 150 (open circle with solid line), 100 (open circle with broken line), 50 (brown circle with solid line), or 10 mg/kg (brown circle with broken line), gPTX-L at the dose of 150 mg/kg (open triangle), gPTX-L at the dose of 150 mg/kg with 150 mg/kg trastuzumab (gPTX-L + Tras, open square), naked gPTX at the dose of 100 mg/kg (open diamond), trastuzumab (Tras, closed square), CEP-IL (closed circle), or PBS (cross) was intravenously injected. A, Tumor weights at day 30. B, Changes in body weight. Data are presented as the mean ± S.D. *, *P*<0.05 compared with PBS.

**Figure 8:**
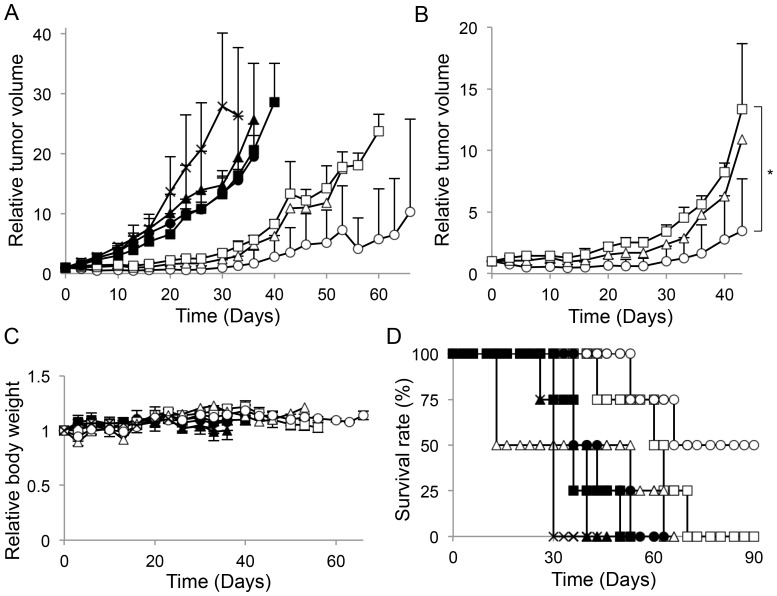
Anticancer efficacy of different gPTX formulations with repeated administration in HT-29 cells tumor-bearing mice. gPTX-IL (open circle with line), gPTX-L (open triangle), gPTX-L with trastuzumab (open square), trastuzumab (closed square), CEP-IL (closed circle), CEP-L (closed triangle), or PBS (cross) was intravenously injected at day 0, 10, and 20. The dose of each administration was 150 mg/kg gPTX. A and B, Changes in tumor volume. C, Changes in body weight. D, Survival curves. Data are presented as the mean ± S.D. The changes of tumor volume and body weight in the mice administered with gPTX-L do not show S.D. after day 10. The *P* value shown compared with gPTX-IL and gPTX-L with trastuzumab treated group at day 43 (n = 4). *, *P*<0.05.

The *in vivo* antitumor effects of different gPTX formulations were evaluated in tumor-bearing mice that had been transplanted with HT-29 cells. Although a single administration of gPTX-L with/without trastuzumab at a gPTX-equivalent dose of 150 mg/kg did not suppress tumor growth, gPTX-IL decreased tumor weight in a dose-dependent manner and the gPTX-equivalent doses of both 100 and 150 mg/kg of gPTX-IL significantly suppressed tumor growth without affecting body weight (Fig. 7). We further evaluated the antitumor effect of repeated administration of the total dose of 450 mg/kg (Fig. 8). gPTX-IL showed the most effective suppression of tumor growth without significant loss of body weight. The mice treated with gPTX-IL exhibited the longest 50% survival period more than 90 days after the first administration.

## Discussion

In this study, the ability of gPTX to stabilize tubulin polymerization was 90% of that of PTX (Fig. 1). Although this level of activity should be sufficient to inhibit cell growth and proliferation, the IC_50_ of gPTX was 3–10-fold higher than that of PTX (Fig. 5A). This apparent discrepancy between the tubulin stabilization activity and the observed inhibition of cell growth could be due to the hydrophilicity of gPTX, since the efficiency of penetration into the lipid bilayer of the cell membrane depends on hydrophobicity. As a result, gPTX exhibited weaker cytotoxicity than PTX. In this context, gPTX may be safer than PTX with respect to the potential for side effects when administered *in vivo*. A suitable formulation of gPTX to efficiently penetrate the cell membrane should enhance the availability as an anticancer drug more effectively than PTX. It appears that the immunoliposome formulation should be applicable to confer gPTX with penetrability. Since we found that gPTX was soluble in 40% EG and CEP, we first attempted direct encapsulation of gPTX into liposomes in these solvents, but the EE was not practical to prepare enough gPTX-L for clinical application (Table S1).

The remote loading method is a typical method to facilitate efficient encapsulation of drugs into the inner water core of liposomes. PEGylated liposome-encapsulated doxorubicin, known as Doxil, is prepared by the ammonium sulfate gradient remote loading method [18]. The application of a pH gradient method was developed for the encapsulation of a derivative of docetaxel modified with a weak base group [23]. However, this method is not considered suitable for the encapsulation of uncharged drugs such as taxanes. Hence, we developed an approach to use the different solubilities of gPTX in solvents in the remote loading procedure. We developed a novel remote loading method using a solubility gradient between 40% EG and CEP to achieve efficient encapsulation of gPTX and PTX into liposomes (Fig. 4A–C). When gPTX dissolved in 40% EG was added to 40% EG-encapsulated liposomes, EE was <10%. This result suggested that the solubility gradient acted as a driving force for active encapsulation. This is the first report of an efficient method for encapsulating uncharged molecules such as PTX and its derivative into liposomes by a remote loading method.

PTX-L was labile even when maintained at 4°C, and TEM image revealed that it existed as a nonliposomal formulation after 4 days of incubation at 4°C. This is likely because sufficient amounts of PTX accumulated in the hydrophobic space in the lipid bilayer, resulting in the breakdown of the liposomes and subsequent release of free PTX. This might be the reason why the amounts of PTX encapsulated into liposomes is limited, although liposome encapsulated-PTX in which PTX is inserted into the lipid bilayer has been demonstrated [17]. In contrast, gPTX-L prepared by remote loading displayed almost 100% retention rate during 4 weeks of incubation at 4°C. These results suggested that most gPTX is efficiently encapsulated into the inner water core of liposomes by the solubility gradient method. The hydrophilic character of gPTX compared to PTX could explain the good retention of gPTX in the inner water core of liposomes.

Although Cremophor EL and ethanol are toxic, the commercial formulation of PTX, Taxol, contains both as the solvent. Thus, the development of liposomal PTX has been an important issue for the use of this drug as an effective anticancer agent to avoid the toxicity of the solvent and improve the efficacy of Taxol. In the solubility gradient method, the solvent is directly encapsulated into the liposomes. CEP-L should have fewer side effects than free CEP because the compounds should be simultaneously delivered to tumor tissues. This hypothesis was confirmed in the *in vivo* experiment (Figs. 8 and S4). CEP-L did not cause serious side effects such as loss of body weight *in vivo*, whereas free CEP was toxic in the assessment for acute toxicity (Fig. S2).

To enhance the therapeutic effect of gPTX-L, we designed immunoliposomes to target HER2 by displaying trastuzumab on the surface of the liposomes. An *in vitro* cytotoxic assay after 72 h of drug exposure revealed that gPTX-L exhibited enhanced cytotoxicity compared with gPTX. This result suggests that large amounts of gPTX simultaneously internalize into the cells with the liposomal formulation. The IC_50_ of gPTX-IL was almost similar to that of gPTX-L, even in HER2-overexpressing cancer cells. However, gPTX-IL effectively suppressed the growth of HER2-overexpressing cancer cells at the shortest IT_50_ among the formulations. Thus, these results suggest that gPTX-IL efficiently targets and internalizes into HER2-overexpressing cancer cells via HER2-mediated endocytosis [14], [24].

Liposomal formulation of PTX decreased the side effects of naked PTX because PTX is not bioavailable until PTX is released from liposomes [16], [25]. We also demonstrated that liposomal formulation of gPTX decreased the side effects of naked gPTX (Fig. S2). The naked gPTX at the dose of 150 mg/kg was lethal to mice and at both doses of 100 and 150 mg/kg gPTX showed significant loss of body weights. However, the mice treated with gPTX-L at the same doses of naked gPTX dramatically alleviated the acute toxicity. gPTX-IL at the dose of 150 mg/kg also decreased the side effects of gPTX such as significant loss of body weight and lethality just as gPTX-L did (Fig. 6). PTX encapsulated into liposomes have showed large amounts of PTX accumulate into the liver of the mice [13], [26], [27]. Reticuloendothelial system such as the liver is a major mechanism for clearance of the liposomes [28]. Drugs encapsulated into liposomes should be excreted via liver tissue together with bile acids. Once PTX is excreted into intestine it will not be reabsorbed due to P-glycoprotein as well as the enzymatic degradation [29]. Since PTX itself is considered to be metabolized predominantly in the liver with cytochrome P450 [30]. Cytochrome P450 enzymatically detoxifies PTX while highly excess dose of PTX should still be lethally toxic. Taking these into consideration, gPTX in liposomes should be excreted into intestine where P-glycoproteins prevent reabsorption. On the other hand, naked gPTX is metabolized by cytochrome P450 in the liver while the excess amount of gPTX would affect the whole of the body. Therefore, it is conceivable that liposomal formulation of gPTX alleviates the side effects of gPTX.

Finally, we evaluated the antitumor effects of gPTX-L and gPTX-IL *in vivo*. While treatment of mice with naked gPTX at the dose of 100 mg/kg significantly decreased body weight, treatment with gPTX-L and gPTX-IL did not significantly affect body weight even when the gPTX-equivalent dose was 150 mg/kg, which was lethal when gPTX dissolved in CEP was directly injected into the mice. Although gPTX-L at the gPTX-equivalent dose of 150 mg/kg did not suppress tumor growth, gPTX-IL at the equivalent dose decreased tumor volume and effectively suppressed tumor growth in HT-29 tumor-bearing BALB/c-nu/nu and ICR-nu/nu mice in single administration experiments (Figs. 7 and S4). We further evaluated antitumor efficiency with repeated administration at the total dose of 450 mg/kg gPTX. Both gPTX-L and -IL effectively inhibited tumor growth. Furthermore, gPTX-IL exhibited the most effective antitumor activity without side effects, such as a loss of body weight (Fig. 8). We confirmed that liposomes accumulated at the tumor site in HT-29 tumor-bearing mice via EPR effects, and trastuzumab-displaying immunoliposomes exhibited a longer retention time at the tumor site than non-targeting liposomes (Fig. S3). Immunoliposomes efficiently internalize into cancer cells at the tumor site while non-targeting liposomes localize in the stroma of tumor tissue [31]. In this regard, gPTX-IL effectively internalized into the tumor cells and strongly inhibited tumor growth. On the other hand, gPTX-L did not exhibit same antitumor activity as gPTX-IL because non-targeting liposomes did not have a sufficient retention time to affect tumor growth.

Nanoparticle-based PTX formulations, Abraxane (albumin-bound PTX) and Lipusu (liposome-encapsulated PTX), have been commercially and clinically available. The 50% lethal dose (LD_50_) of each drug in mice was reported at the PTX-equivalent dose of 47 and 70 mg/kg, respectively [32], [33]. The LD_50_ of our gPTX-L was more than 150 mg/kg of gPTX. Taking into account the molecular weights and tubulin polymerization assay results, the dose of 150 mg/kg of gPTX is equivalent to the dose of 100 mg/kg of PTX. Furthermore, gPTX-IL was also injected at the gPTX-equivalent dose of 150 mg/kg in single administration experiments. Yang et al. previously reported trastuzumab-displaying and PTX encapsulating liposomes [12], [14]. The antitumor activity of their PTX-immunoliposomes was evaluated at the PTX-equivalent total dose of 22.5 mg/kg (three administrations of PTX equivalent of 7.5 mg/kg with a 4-day interval) [12]. The PTX-equivalent dose of our gPTX-IL in antitumor effect of the repeated administration was estimated to be 13-fold higher than that of their PTX-immunoliposomes. Our gPTX-IL thus could be used for drug formulations to enable administration with high effective anticancer activity in patients with HER2 overexpressing cancer cells.

Together our results show that gPTX was efficiently encapsulated into liposomes via the novel encapsulation strategy including a solubility gradient. Immunoliposomes encapsulated gPTX successfully exhibited efficient anticancer activity against HER2-overexpressing cancer cells *in vitro* and *in vivo*. Thus, we propose gPTX-IL should be an excellent candidate as a targeted drug delivery system.

## Conclusions

gPTX generated by coupling a glucose moiety at the 7-OH residue of PTX allowed encapsulation of the drug into liposomes with high efficiency without a significant decrease in its ability to stabilize tubulin polymerization. The cytotoxic activity of gPTX was significantly less than that of PTX, suggesting that the hydrophilic character of gPTX could prevent the molecule from permeating through the lipid bilayer of cells. Encapsulation of gPTX into liposomes with high efficiency was successfully achieved by the novel remote loading strategy under a solubility gradient between CEP and 40% EG. This strategy should enable the preparation of sufficient quantities of both gPTX-L and gPTX-IL for practical therapy. Notably, the cytotoxicity of gPTX-IL was dependent on the exposure time, suggesting that the potential of immunoliposomes should be high in targeting cell surface antigens. As expected, gPTX-IL strongly inhibited tumor growth of HER2-overexpressing cancer cells with less side effects *in vivo*. Taken together, the immunoliposomes containing effective amounts of gPTX should be a promising formulation of anticancer drugs for targeted drug delivery systems with gPTX.

## Supporting Information

Figure S1 **TEM image of gPTX-IL.** The formulation was observed with TEM.
(TIF)

Figure S2 **Acute toxicity of gPTX and gPTX-L** ***in vivo*** **.** gPTX (open circle) or gPTX-L (closed circle) at a concentration of 150 mg/kg, gPTX (open triangle) or gPTX-L (closed triangle) at a concentration of 100 mg/kg, CEP (open square), or PBS (cross) was intravenously injected into 6-week old female BALB/c mice. A, Changes in body weight. B, Survival rate. Data are presented as the mean ± S.D. (n = 4). gPTX at the concentration of 150 mg/kg and CEP are not shown S.D. after day 1.
(TIF)

Figure S3 **Distribution of HSA-Cy5.5 in tumor-bearing ICR-nu/nu mice transplanted with HT29 cells.** When the tumor volume reached 100–200 mm^3^, HSA-Cy5.5-IL, HSA-Cy5.5-L, free HSA-Cy5.5, and HSA as a control were intravenously injected into mice. Cy5.5 fluorescence was detected 2, 4, 6, 12, 24, and 48 h after injection. Each arrow indicates the location of tumor tissue.
(TIF)

Figure S4 **Anticancer efficacy of different gPTX formulations in tumor-bearing ICR-nu/nu mice transplanted with HT-29 cells.** When the tumor volume reached 50–200 mm^3^, gPTX-IL (open circle), gPTX-L (open triangle), CEP-IL (closed circle), CEP-L (closed triangle), trastuzumab (closed square), or PBS as a control (cross) was intravenously injected at a dose of 150 mg/kg gPTX on day 0. A and B, Changes in tumor volume. C, Changes in body weight. Data are presented as the mean ± S.D. (n = 4). *, *P*<0.05 at day 30 for gPTX-IL compared with the other treatments.
(TIF)

Table S1 **Encapsulation efficiency (EE) and loading efficiency (LE) of PTX-L or gPTX-L in a direct encapsulation method with 40% EG and CEP at the maximal concentration.**
(DOCX)

Table S2 **Cytotoxicity of different PTX formulations in HER2-overexpressing cancer cells (HT-29 and SK-BR-3) and HER2 low-expressing cancer cells (MDA-MB-231).**
(DOCX)

Methods S1 (DOCX)
