# Supplementary figures and images for: Efficient Drug Delivery of Paclitaxel Glycoside: A Novel Solubility Gradient Encapsulation into Liposomes Coupled with Immunoliposomes Preparation
Source: PLoS One. 2014 Sep 29;9(9):e107976. doi: 10.1371/journal.pone.0107976 (PMC4180071; doi:10.1371/journal.pone.0107976)

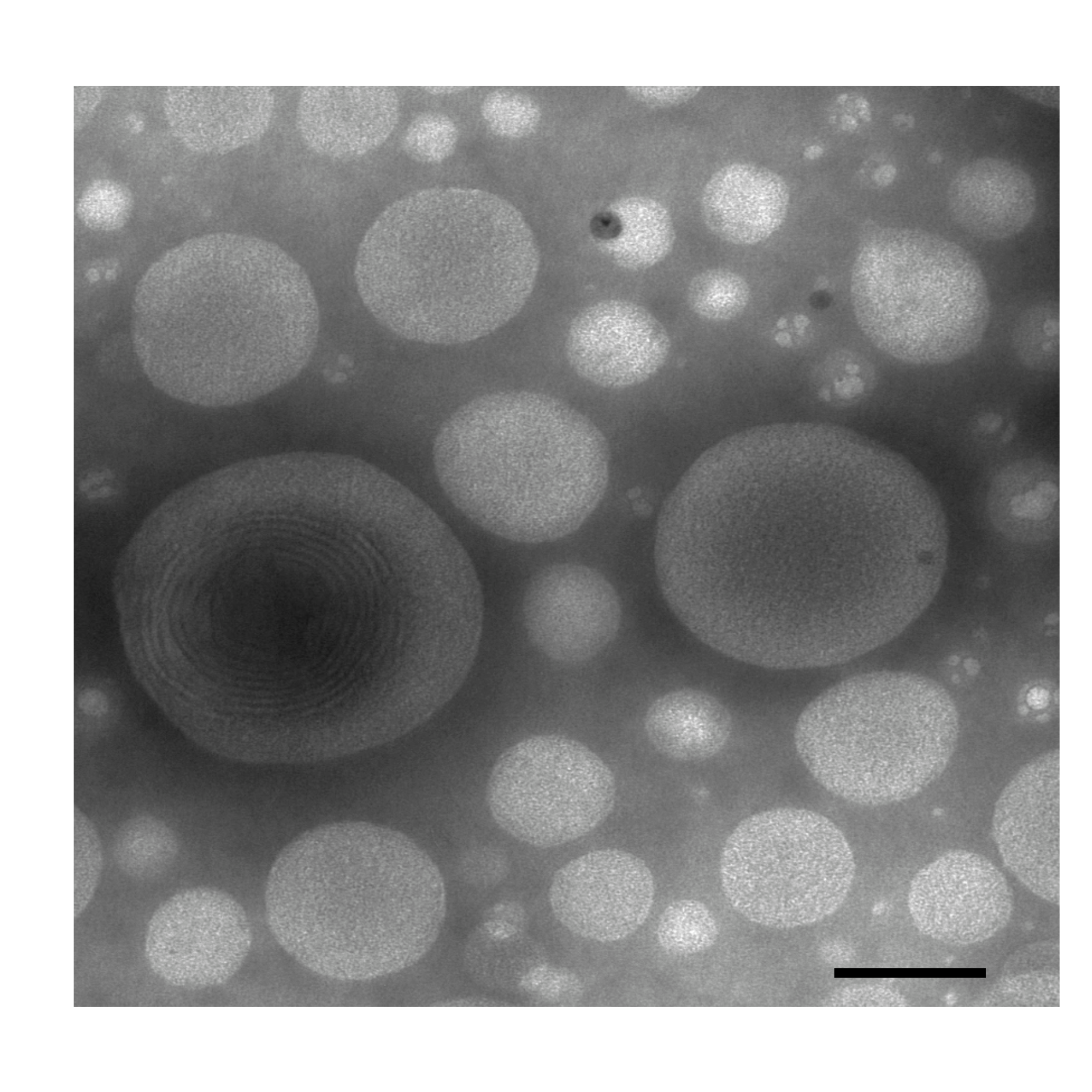

Supplement: Figure S1 — TEM image of gPTX-IL. The formulation was observed with TEM. (TIF) [file pone.0107976.s001.tif]

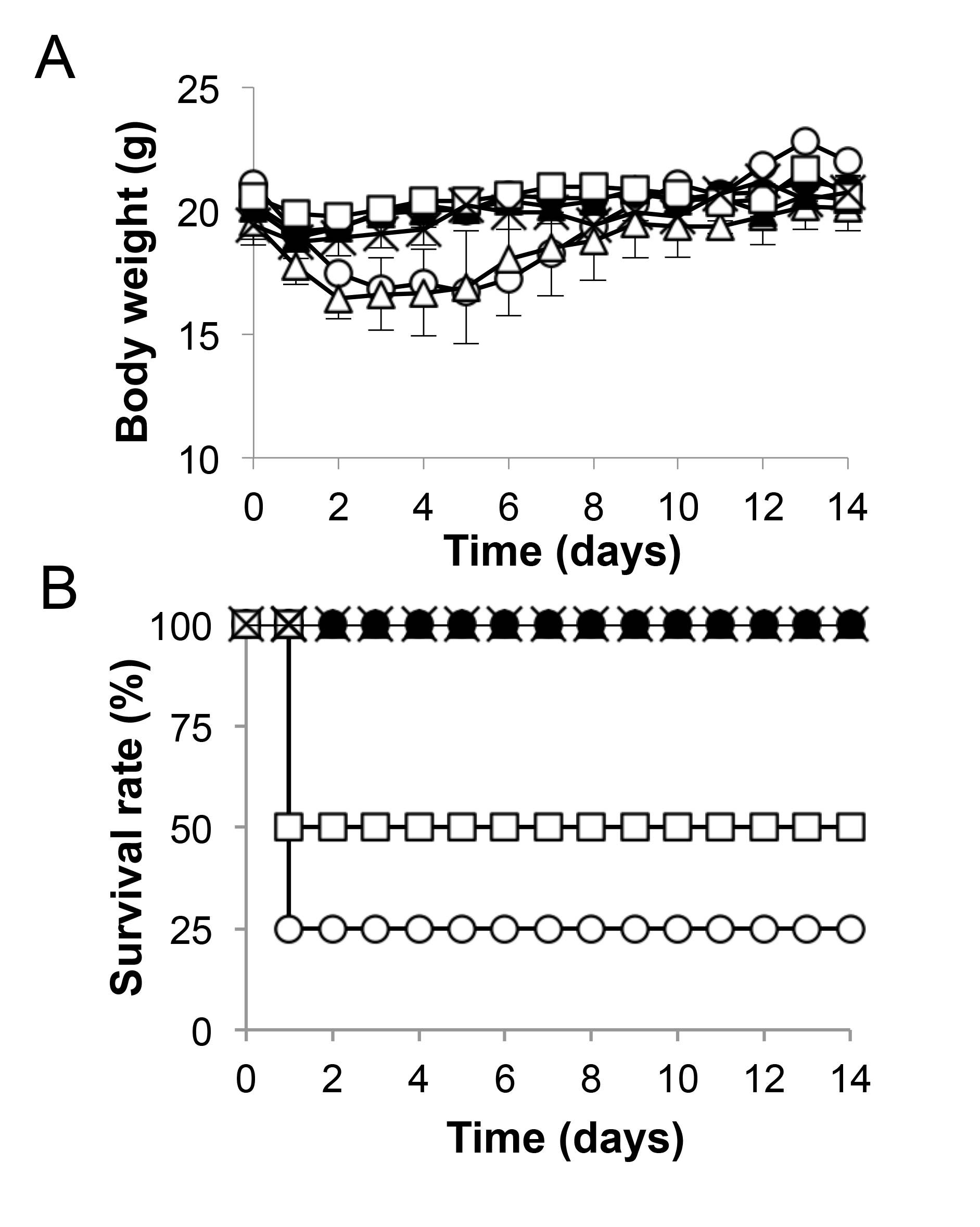

Supplement: Figure S2 — Acute toxicity of gPTX and gPTX-L in vivo . gPTX (open circle) or gPTX-L (closed circle) at a concentration of 150 mg/kg, gPTX (open triangle) or gPTX-L (closed triangle) at a concentration of 100 mg/kg, CEP (open square), or PBS (cross) was intravenously injected into 6-week old female BALB/c mice. A, Changes in body weight. B, Survival rate. Data are presented as the mean ± S.D. (n = 4). gPTX at the concentration of 150 mg/kg and CEP are not shown S.D. after day 1. (TIF) [file pone.0107976.s002.tif]

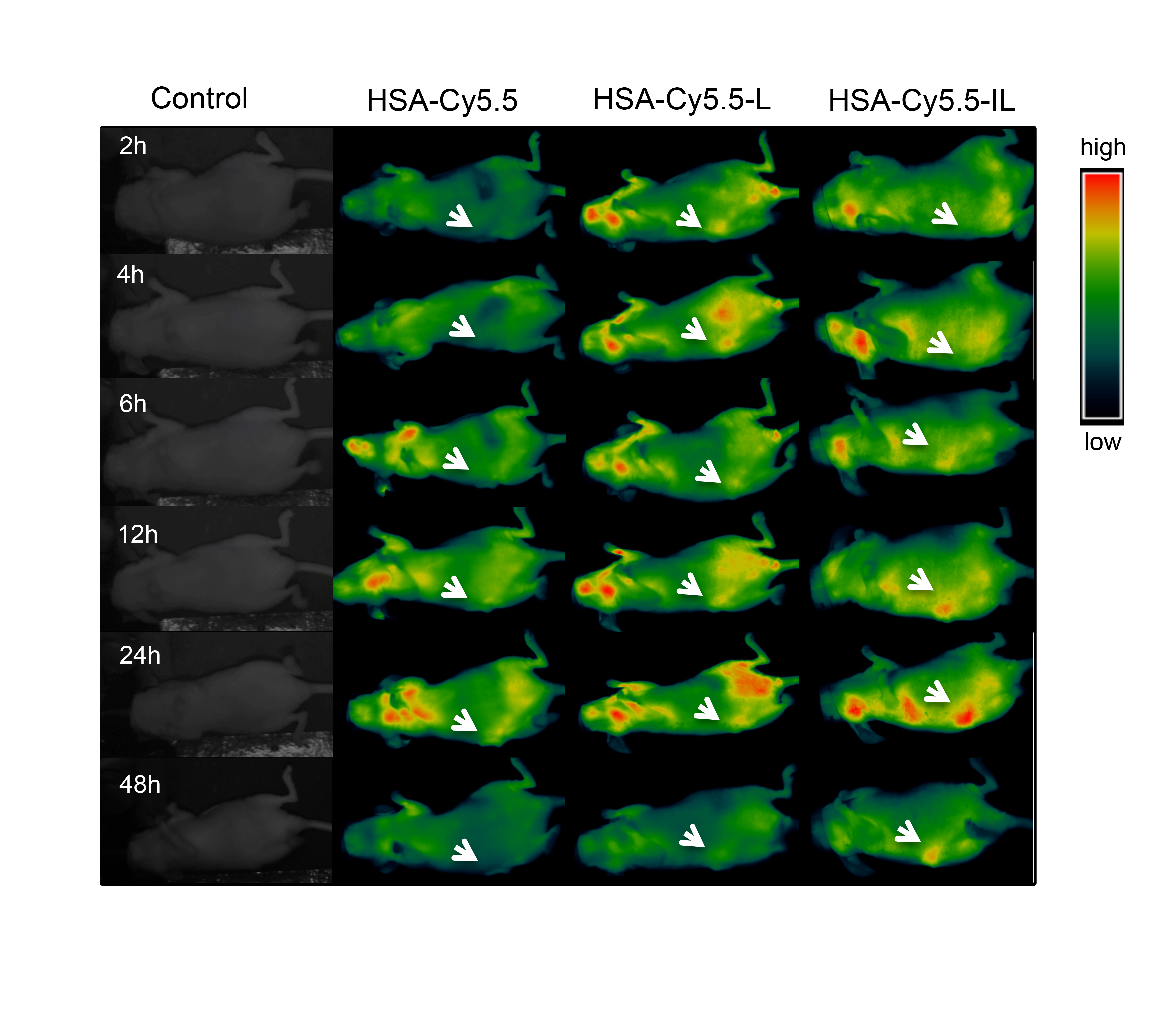

Supplement: Figure S3 — Distribution of HSA-Cy5.5 in tumor-bearing ICR-nu/nu mice transplanted with HT29 cells. When the tumor volume reached 100–200 mm3, HSA-Cy5.5-IL, HSA-Cy5.5-L, free HSA-Cy5.5, and HSA as a control were intravenously injected into mice. Cy5.5 fluorescence was detected 2, 4, 6, 12, 24, and 48 h after injection. Each arrow indicates the location of tumor tissue. (TIF) [file pone.0107976.s003.tif]

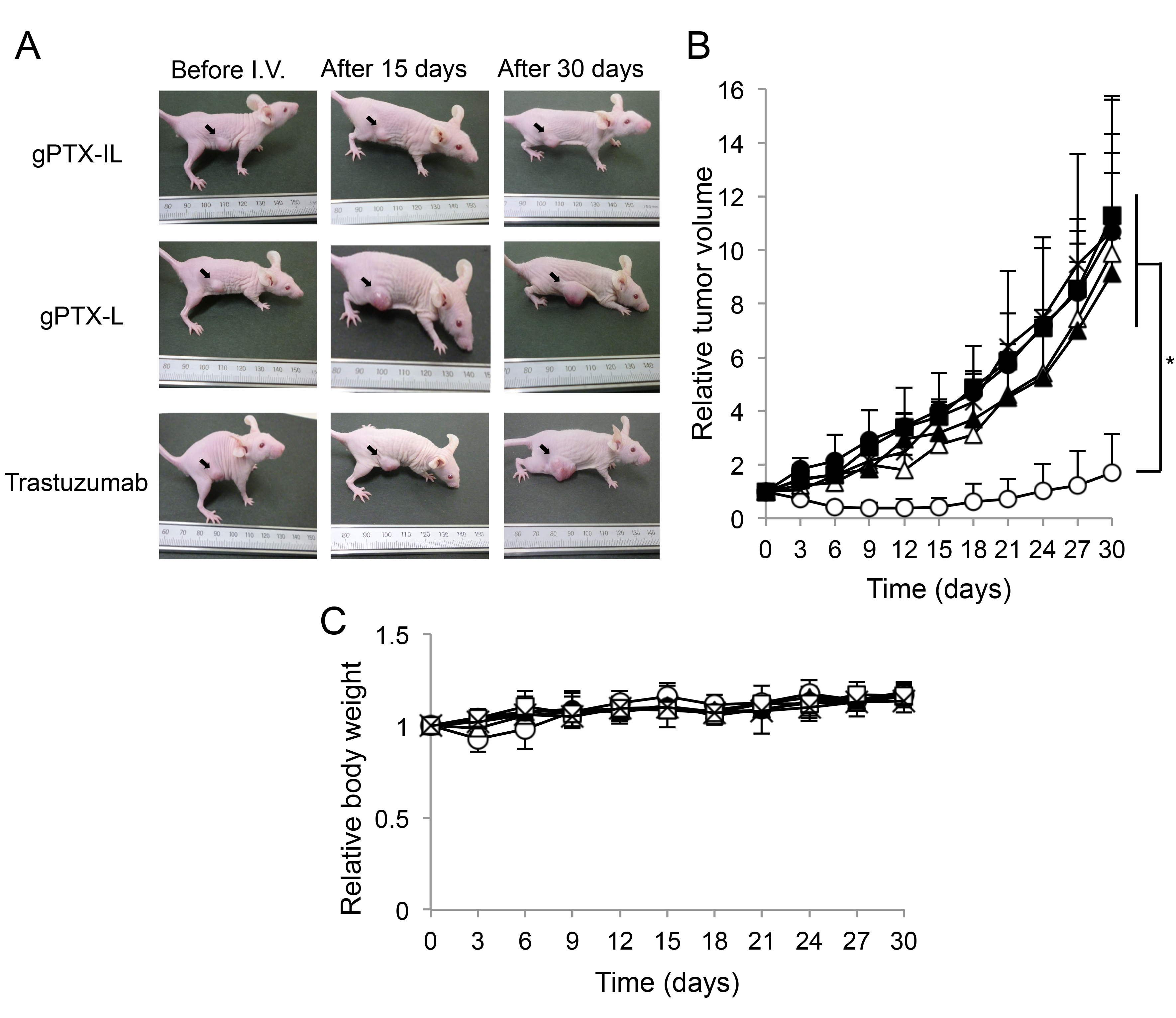

Supplement: Figure S4 — Anticancer efficacy of different gPTX formulations in tumor-bearing ICR-nu/nu mice transplanted with HT-29 cells. When the tumor volume reached 50–200 mm3, gPTX-IL (open circle), gPTX-L (open triangle), CEP-IL (closed circle), CEP-L (closed triangle), trastuzumab (closed square), or PBS as a control (cross) was intravenously injected at a dose of 150 mg/kg gPTX on day 0. A and B, Changes in tumor volume. C, Changes in body weight. Data are presented as the mean ± S.D. (n = 4). *, P<0.05 at day 30 for gPTX-IL compared with the other treatments. (TIF) [file pone.0107976.s004.tif]
