# Supplementary material for: Efficient Drug Delivery of Paclitaxel Glycoside: A Novel Solubility Gradient Encapsulation into Liposomes Coupled with Immunoliposomes Preparation
Source: PLoS One. 2014 Sep 29;9(9):e107976. doi: 10.1371/journal.pone.0107976 (PMC4180071; doi:10.1371/journal.pone.0107976)
Supplement: Table S1 — Encapsulation efficiency (EE) and loading efficiency (LE) of PTX-L or gPTX-L in a direct encapsulation method with 40% EG and CEP at the maximal concentration. (DOCX) [file pone.0107976.s005.docx]

Table S1. Encapsulation efficiency (EE) and loading efficiency (LE) of PTX-L or gPTX-L in a direct encapsulation method with 40% EG and CEP at the maximal concentration.

|  | Drug Solvent | EE (%) | LE (%) |
| --- | --- | --- | --- |
| PTX-L | 40% EG | N.D.^*^ | N.D.^*^ |
|  | CEP | 12.1 | 1.2 |
| gPTX-L | 40% EG | 0.4 | 0.03 |
|  | CEP | 17.6 | 13.7 |

**, N.D. means not determined*
