# Supplementary material for: Efficient Drug Delivery of Paclitaxel Glycoside: A Novel Solubility Gradient Encapsulation into Liposomes Coupled with Immunoliposomes Preparation
Source: PLoS One. 2014 Sep 29;9(9):e107976. doi: 10.1371/journal.pone.0107976 (PMC4180071; doi:10.1371/journal.pone.0107976)
Supplement: Table S2 — Cytotoxicity of different PTX formulations in HER2-overexpressing cancer cells (HT-29 and SK-BR-3) and HER2 low-expressing cancer cells (MDA-MB-231). (DOCX) [file pone.0107976.s006.docx]

Table S2. Cytotoxicity of different PTX formulations in HER2-overexpressing cancer cells (HT-29 and SK-BR-3) and HER2 low-expressing cancer cells (MDA-MB-231).

|  | HT-29 | | | SK-BR-3 | | | MDA-MB-231 | | |
| --- | --- | --- | --- | --- | --- | --- | --- | --- | --- |
|  | IC_50_  (nM) | IC_100_ (nM) | IT_50_  (h) | IC_50_  (nM) | IC_100_  (nM) | IT_50_  (h) | IC_50_  (nM) | IC_100_ (nM) | IT_50_  (h) |
| PTX | 1.3 ± 0.5 | 10 | 7.6 ± 0.8 | 5.5 ± 1.3 | 30 | 9.1 ± 0.6 | 3.1 ± 0.5 | 30 | 3.3 ± 0.5 |
| gPTX | 11.0 ± 0.8 | 50 | 9.4 ± 1.6 | 18.9 ± 1.1 | 100 | 10.5 ± 2.1 | 7.7 ± 1.3 | 50 | 6.2 ± 2.8 |
| gPTX-L | 7.6 ± 1.3 | 30 | 7.1 ± 0.4 | 6.6 ± 0.9 | 30 | 8.9 ± 1.2 | 4.6 ± 0.6 | 30 | 3.2 ± 0.8 |
| gPTX-IL | 6.7 ± 0.6 | 30 | 4.7 ± 0.7 | 5.3 ± 0.7 | 30 | 6.4 ± 0.8 | 4.9 ± 0.6 | 30 | 3.5 ± 0.7 |

*IC_50_ and IT_50_ are presented as the mean ± S.D. (n = 3).*

*IC_100_ was estimated from the evaluation of cytotoxicity.*
