# Supplementary material for: Efficient Drug Delivery of Paclitaxel Glycoside: A Novel Solubility Gradient Encapsulation into Liposomes Coupled with Immunoliposomes Preparation
Source: PLoS One. 2014 Sep 29;9(9):e107976. doi: 10.1371/journal.pone.0107976 (PMC4180071; doi:10.1371/journal.pone.0107976)
Supplement: Methods S1 — (DOCX) [file pone.0107976.s007.docx]

**Methods S1.**

**Direct encapsulation of PTX/gPTX into liposomes with 40% EG or CEP at the maximal concentration.**

Liposomes composed of DPPC, DPPG, and Chol at a molar ratio 9:1:2 were prepared using a thin-film hydration method. In brief, DPPC, DPPG, and Chol were dissolved in an organic solvent consisting of chloroform/methanol (9:1 v/v) into an egg flask. The flask was connected to a rotary evaporator, which was maintained at 45°C under aspirator vacuum. The resulting lipid film was left overnight under vacuum to remove the remaining organic solvent. The completely dehydrated lipid film was suspended in 40% EG or CEP containing PTX/gPTX at the maximal concentration. The ratio of PTX/gPTX to initial lipids/Chol was at 0.06 (w/w) in 40% EG and 0.1 or 1.1 (w/w) in CEP. After residual PTX or gPTX was removed by washing the liposomes with PBS followed by ultrafiltration at 12,000 × *g* for 20 min for five times, PTX- or gPTX-L was collected. EE was evaluated by reverse-phase HPLC analysis.

**Assessment of immunoliposome distribution *in vivo*.**

To evaluate the targeting effect of immunoliposomes *in vivo*, we prepared liposomes encapsulating human serum albumin (HSA) conjugated with Cy 5.5. In brief, 1 mL of 10 mg/mL HSA in 0.1 M sodium carbonate buffer (pH 9.3) was dissolved in a vial of Cy5.5 Monofunctional Dye (GE Healthcare) and incubated for 30 min at 25°C. HSA conjugated with Cy5.5 (HSA-Cy5.5) was purified by G25 gel column chromatography. The liposomes and immunoliposomes were prepared as described in the section “Preparation of immunoliposomes containing gPTX.” When the volume of tumors of HT-29 cell-bearing ICR-nu/nu mice reached 100–200 mm^3^, liposomes and immunoliposomes encapsulating HSA-Cy5.5 were intravenously injected via the tail vein. The fluorescence of Cy5.5 was detected by a Lumazone *in vivo* imaging system (Nippon Roper, Tokyo, Japan) at 2, 4, 6, 12, 24, and 48 h after administration.
